# High-throughput field phenotyping using hyperspectral reflectance and partial least squares regression (PLSR) reveals genetic modifications to photosynthetic capacity

**DOI:** 10.1016/j.rse.2019.04.029

**Published:** 2019-09-15

**Authors:** Katherine Meacham-Hensold, Christopher M. Montes, Jin Wu, Kaiyu Guan, Peng Fu, Elizabeth A. Ainsworth, Taylor Pederson, Caitlin E. Moore, Kenny Lee Brown, Christine Raines, Carl J. Bernacchi

**Affiliations:** a Department of Plant Biology, University of Illinois at Urbana-Champaign, USA; b Carl R Woese Institute for Genomic Biology, University of Illinois at Urbana-Champaign, USA; c Environmental & Climate Science Department, Brookhaven National Laboratory, Upton, New York, USA; d School of Biological Sciences, University of Hong Kong, Pokfulam, Hong Kong; e Department of Natural Resources and Environmental Sciences, University of Illinois at Urbana-Champaign, IL, USA; f National Center of Supercomputing Applications, University of Illinois at Urbana-Champaign, USA; g USDA ARS Global Change and Photosynthesis Research Unit, Urbana, IL, USA; h Department of Biological Sciences, University of Essex, Colchester CO4 3SQ, United Kingdom

**Keywords:** Hyperspectral reflectance, Partial least squares regression (PLSR), Photosynthesis, Leaf nitrogen, Food security, Gas exchange, Spectroscopy

## Abstract

Spectroscopy is becoming an increasingly powerful tool to alleviate the challenges of traditional measurements of key plant traits at the leaf, canopy, and ecosystem scales. Spectroscopic methods often rely on statistical approaches to reduce data redundancy and enhance useful prediction of physiological traits. Given the mechanistic uncertainty of spectroscopic techniques, genetic modification of plant biochemical pathways may affect reflectance spectra causing predictive models to lose power. The objectives of this research were to assess over two separate years, whether a predictive model can represent natural and imposed variation in leaf photosynthetic potential for different crop cultivars and genetically modified plants, to assess the interannual capabilities of a partial least square regression (PLSR) model, and to determine whether leaf N is a dominant driver of photosynthesis in PLSR models. In 2016, a PLSR analysis of reflectance spectra coupled with gas exchange data was used to build predictive models for photosynthetic parameters including maximum carboxylation rate of Rubisco (*V*_*c*,*max*_), maximum electron transport rate (*J*_*max*_) and percentage leaf nitrogen ([N]). The model was developed for wild type and genetically modified plants that represent a wide range of photosynthetic capacities. Results show that hyperspectral reflectance accurately predicted *V*_*c*,max_, *J*_*max*_ and [N] for all plants measured in 2016. Applying these PLSR models to plants grown in 2017 resulted in a strong predictive ability relative to gas exchange measurements for *V*_*c*,max_, but not for *J*_max_, and not for genotypes unique to 2017. Building a new model including data collected in 2017 resulted in more robust predictions, with R^2^ increases of 17% for *V*_*c*,*max*_. and 13% *J*_*max*_. Plants generally have a positive correlation between leaf nitrogen and photosynthesis, however, tobacco with reduced Rubisco (SSuD) had significantly higher [N] despite much lower *V*_*c*,max_. The PLSR model was able to accurately predict both lower *V*_*c*,*max*_ and higher leaf [N] for this genotype suggesting that the spectral based estimates of *V*_*c*,*max*_ and leaf nitrogen [N] are independent. These results suggest that the PLSR model can be applied across years, but only to genotypes used to build the model and that the actual mechanism measured with the PLSR technique is not directly related to leaf [N]. The success of the leaf-scale analysis suggests that similar approaches may be successful at the canopy and ecosystem scales but to use these methods across years and between genotypes at any scale, application of accurately populated physical based models based on radiative transfer principles may be required.

## Highlights

• We built PLS models to predict photosynthesis from hyperspectral reflectance.
• The models work for plants with genetic modification to photosynthesis.
• Predicted photosynthetic capacity is uncoupled from leaf nitrogen.
• PLS photosynthesis models must be built with representation of all genetic material.

## 1 Introduction

Projected population increases, rising global affluence, and mounting pressures from a changing global climate necessitate improvements to global food supply (Tilman et al., 2009; Foley et al., 2011). Yield increases brought about from the ‘Green Revolution’ have plateaued over the last two decades for many crop species (Fischer and Edmeades, 2010; Parry et al., 2010), requiring novel strategies to realize further gains in productivity. Improving photosynthetic potential likely remains the best strategy to increase crop production (Monteith and Moss, 1977; Zhu et al., 2010; Ort et al., 2015), potentially without the need for additional fertilizer and pesticide that were critical to support yield increases associated with the Green Revolution (Long et al., 2006; Evans, 2013).

Despite being one of nature's most conserved processes, photosynthesis has a staggering number of component inefficiencies (Long et al., 2006; Evans, 2013). These inefficiencies inspire current research efforts to improve crop yields through manipulating photosynthetic pathways (Ort et al., 2015; Andralojc et al., 2018) and exploiting natural variation in photosynthetic rates (Lawson et al., 2012; Meacham et al., 2016). Regardless of the means of improvement, the ability to non-destructively sample phenotypic variation in photosynthetic capacity among tens to hundreds of thousands of plants representing genotypic variation within a reasonable time presents a significant phenotyping challenge (Furbank and Tester, 2011).

Remote sensing communities have long used spectral vegetation indices to estimate leaf and canopy properties at the ecosystem scale (Peñuelas et al., 1994; Curran et al., 1997; Martin and Aber, 1997; Ustin et al., 2004; Asner and Martin, 2008). More recently, spectral detection of emission at discreet wavebands corresponding to solar induced fluorescence (SIF) (Frankenberg and Berry, 2018) have been used as a functional proxy for gross primary productivity (GPP) of natural and forest ecosystems (Guanter et al., 2007; Meroni et al., 2009; Yang et al., 2018; Guan et al., 2016) and crops (Miao et al., 2018; Yang et al., 2018). While SIF is valuable in the context of inferring GPP, it does not provide insights into the underlying photosynthetic mechanisms, i.e., maximum Rubisco carboxylation (*V*_*c*,*max*_) and maximum electron transport rate (*J*_*max*_), that are used as indicators of photosynthetic capacity and to model vegetation at leaf- to ecosystem-scales (e.g., Bernacchi et al., 2013; Bagley et al., 2015a, Bagley et al., 2015b). Improving photosynthetic productivity for increased global crop yields requires techniques to quantify these parameters, yet traditional methods rely on leaf sampling and analysis under laboratory conditions or using in-field gas exchange systems (Long and Bernacchi, 2003). These provide a wealth of photosynthetic information but are costly and time intensive. However, spectroscopy techniques coupled with regression analysis (Serbin et al., 2012, Serbin et al., 2014) have been used to screen for germplasms among cropping species with the highest photosynthetic potential (Ainsworth et al., 2014; Yendrek et al., 2016; Silva-Perez et al., 2017). Because of the challenges with gas exchange measurements, remote sensing of intensive agricultural regions of the planet is limited by ground-truth data and the potential for genetically modified crops to increase in areal extent suggests methods are required that quantify *V*_*c*,max_ and *J*_*max*_ over larger spatial areas, beyond what is capable with traditional techniques.

Statistical approaches that link spectral reflectance patterns with ‘ground-truth’ measurements from traditional techniques have potential to significantly decrease sampling time by orders of magnitude. The partial least squares regression (PLSR) model (Wold et al., 2001), which relates two data matrices using a linear multivariate model to predict plant properties of interest, is increasingly used for this purpose. This approach has been applied to rapidly collected leaf reflectance spectra and used to predict key photosynthetic parameters in Aspen and Cottonwood trees (Serbin et al., 2012), soybean exposed to ozone treatments (Ainsworth et al., 2014), wheat (Silva-Perez et al., 2017) and maize (Yendrek et al., 2016; Heckmann et al., 2017).

Before combined gas exchange and aerial spectroscopic techniques can be employed to quantify ecosystem function to feed ecosystem models or to be used as breeding tools, the combination of these techniques must be tested at scales that eliminate confounding factors. Leaf-scale analysis using spectral sensors with an artificial light source on field grown plants, provides an ideal testbed for spectroscopic techniques as it removes many of the issues with spectral measurements at larger scales. While hyperspectral analysis has focused on inter- and intra-specific variation in photosynthetic potential (Ainsworth et al., 2014; Silva-Perez et al., 2017; Yendrek et al., 2016; Heckmann et al., 2017), there is considerable uncertainty whether this technique can be applied to plants in which the photosynthetic machinery has been genetically modified. Genetic modifications can range from optimizing concentrations of existing proteins (e.g., Driever et al., 2017; López-Calcagno et al., 2019) to metabolic engineering of novel pathways (e.g., South et al., 2019). As the underlying mechanisms measured with PLSR techniques remain elusive, artificially altering the amount, or introducing novel proteins and pathways may challenge the predictive ability of these models. Estimations of photosynthetic physiology have also been attributed to leaf nitrogen content ([N]) as calculated from key light absorption pigments in the reflectance spectra (Kattge et al., 2009; Rogers, 2014; Walker et al., 2014; Dechant et al., 2017). This also questions whether altered photosynthetic potential independent of changes in leaf [N] would lead to an inability of spectroscopic analysis of photosynthetic potential in genetically modified plants. If leaf-scale spectroscopic techniques are unable to accurately identify natural and/or imposed variation in photosynthetic potential among a diverse collection of crop genotypes, then it would question the potential for this technique at larger spatial scales relevant for breeding and modeling purposes.

In this study a PLSR model is used to predict photosynthetic capacity from leaf hyperspectral reflectance in field grown wild-type cultivars and genetically modified lines of *Nicotiana tabacum* (tobacco) over multiple time periods over two growing seasons. Specifically, the objectives are to determine whether (1) PLSR based spectral models predict photosynthetic capacity in genetically modified plants, (2) the PLSR model can be applied across growing seasons, and (3) PLSR can predict *V*_*c*,max_ and *J*_*max*_ independent of leaf nitrogen. Tobacco was chosen as a model crop species to test the effectiveness of modifications to the photosynthetic pathway based on the ease of genetic transformation, short growing seasons, and large number of seeds produced (Kromdijk et al., 2016). This allows rapid field trial testing prior to insertion of promising modifications into staple food crops. These objectives were tested on wild type cultivars exhibiting natural variation in photosynthetic capacities and genotypes genetically modified to present increased and decreased photosynthetic potential (Table 1).

**Table 1:** *Nicotiana tabacum* genotypes used in this study and brief description of transgenic modification, with reference for detailed description of transformation.

Year(s) grown	Genotype	Transgene	Transgene expected function
2016 & 2017	Petite Havana	None (WT)	n/a
2016 & 2017	Samsun	None (WT)	n/a
2016 & 2017	Mammoth	None (WT)	n/a
2016	SFX	Overexpressed photosynthetic carbon reduction cycle enzymes, background: Samsun (Simkin et al., 2015)	Improved photosynthetic capacity, due to increased carbon reduction enzymes.
2016 & 2017	Single Rubsico Knockdown (SSuS)	Rubisco small subunit antisense. 40% of WT Rubisco, background: W38 (Hudson et al., 1992)	Reduced photosynthetic capacity, due to reduced Rubisco
2016 & 2017	Double Rubisco Knockdown (SSuD)	Rubisco small subunit antisense. 10% of WT Rubisco, background: W38 (Hudson et al., 1992)	Reduced photosynthetic capacity, due to reduced Rubisco
2017	200–8	Insertion of two transgenic genes expressing the enzyme Glycolate dehydrogenase and Malate synthase as an alternative pathway to native photorespiration, background: Petite Havana (South et al., 2019)	Increased photosynthetic capacity, by reduction of energy loss associated with photorespiration.
2017	43-OE	Increased PsbS mRNA levels from transformation with *Nicotiana benthamiana* Psbs coding sequence and 35S promoter, background: Petite Havana (Głowacka et al., 2016, Głowacka et al., 2018)	Increased photosynthetic capacity, due to increase in the electron transport metabolite pools.
2017	4-KO	Decreased PsbS mRNA levels from transformation with *Nicotiana benthamiana* Psbs coding sequence and 35S promoter, background: Petite Havana (Głowacka et al., 2016, Głowacka et al., 2018)	Reduced photosynthetic capacity, due to decreased electron transport metabolite pools.

## 2 Methods

### 2.1 Plant material

In 2016, six genotypes consisting of three transgenic and three wild type lines of *Nicotiana tabacum* (Table 1) were grown under field conditions at the University of Illinois Energy Farm Facility in Urbana, Illinois (40°03′46.4″N 88°12′25.4″W, 215 m above sea level). Genotypes were chosen to exhibit variation in photosynthetic capacity using the three wild type cultivars representing different relative growth rates, two transgenic Rubisco antisense lines with reduced photosynthetic capacity (Hudson et al., 1992), and one transgenic with overexpression of photosynthetic carbon reduction cycle enzymes to increase photosynthetic capacity (Simkin et al., 2015). Plants were germinated in greenhouse conditions and transplanted to the field at the four-leaf stage. High levels of ESN Smart Nitrogen (310 kg/ha, equating to ~150 ppm soil concentration) were applied to the field site two weeks prior to transplanting. A biological pesticide *Bacillus thuringiensis v. kurstaki* (54%) (DiPel PRO), was applied to the prepared field site five days prior to transplant and at biweekly intervals thereafter to control for tobacco pests. A broad action herbicide, Glyphosate-isopropylammonium (41%) (Killzall; VPG) (15 l at 70 g/l) was applied to all plots once, two days prior to transplanting. Irrigation was provided as needed to eliminate water limitation throughout growth. The experiment consisted of four replicated plots of each genotype with 36 plants per plot arranged in a 6 × 6 grid and spaced 0.38 m apart.

In 2017, the SFX genotype was removed from the experiment and three transgenic lines were added (Table 1). Plants were grown at the same location following the same protocol as 2016. Two newly introduced lines had either reduced or increased photoprotective quenching capacity, and the third had an alternative photorespiratory pathway relative to the wild-type. Field set up, plot design, pesticide and nutrient application followed the same protocol as 2016.

### 2.2 Leaf reflectance and gas exchange

#### 2.2.1 Leaf reflectance

Leaf spectral reflectance was measured in situ from 400 to 2500 nm using a spectroradiometer (*Fieldspec4*, *Analytical Spectral Devices - ASD*, *Boulder*, *CO USA*), with spectral resolution of 3 nm in the visible and NIR (350–1000 nm) and 8 nm in shortwave-infrared (SWIR; 1000–2500 nm). Measurements were made with a leaf clip attached to the fiberoptic cable. The device contains a radiometrically calibrated light source which was standardized for relative reflectance (white reference) prior to each measurement using a spectralon panel. The last fully expanded leaf on each plant was measured, always keeping its natural orientation, avoiding leaf midrib and leaf edges. Each measurement was the mean of 10 scans at 100 ms scanning speed per scan. Six reflectance spectra were recorded using the leaf clip attachment in different regions of the same leaf, and a total of three leaves were sampled per plot.

A spectral splice correction was applied to each spectrum to align the VIS and SWIR sensor to the NIR sensor, and a bias threshold removed spectra with high light levels at 450 nm as a quality control to ensure the leaf clip was properly fastened onto the leaf during each measurement. The six spectra for a single leaf were then averaged to give a mean spectrum per leaf. Spectra from the six samples with a deviation from the mean greater than 2% reflectance were eliminated using the FieldSpectra package in R according to Serbin et al. (2014). Leaves with a remaining number of viable spectra less than 4 repetitions were eliminated from analysis.

Plants were measured at multiple developmental stages to capture a wide variation in maturity and in meteorological conditions for each genotype. Measurements were collected during three date ranges in 2016: June 30 – July 1 (T1), July 19–21 (T2), and August 4–5 (T3). In 2017 measurements were made on four date ranges: June 26–28 (T1), July 6–12 (T2), July 31–August 1 (T3) and August 18 (T4). Measurements were made on clear sky days between 11 am and 2:30 pm local time (Central Daylight Time). Meteorological conditions for measurement periods are summarized in Fig. S1.

#### 2.2.2 Gas exchange

Within 30 min of the spectral measurements, photosynthetic (*A*) vs. intercellular CO_2_ (*C*_*i*_) response curves were collected to determine *V*_*c*,max_ and *J*_*max*_ for each leaf to use as ground-truth training for a PLSR model. Curves were measured on the same leaves as the hyperspectral measurements using a portable leaf gas exchange system (*LI-6400*, *LICOR Biosciences*, *Lincoln*, *NE*, *USA*). Ambient leaf temperature was determined as the mean of three measurements of leaf temperature with a handheld IR gun (*FLIR TG54*, *FLIR® Systems*, *Inc.*, *Wilsonville*, *Oregon*, *USA*). Block temperature on the gas exchange system was set to match this mean leaf temperature prior to each CO_2_ response curve. PAR was set to 1800 μmol m^−2^ s^−1^, and CO_2_ concentrations were adjusted stepwise over a range of 50 to 2000 μmol mol^−1^ in set increments as follows: 400, 200, 50, 100, 300, 400, 600, 900, 1200, 1500, 1800, 2000. Leaves were acclimated to chamber conditions for a minimum of 300 s prior to initiating each *A*/*C*_*i*_ curve and a minimum and maximum wait time of 160 s and 200 s, respectively, was incorporated before triggering each individual measurement. Relative humidity inside the chamber was manually controlled to 65 ± 5% before each curve by adjusting the flow through the desiccant tube integrated into the gas exchange system. *V*_*c*,*max*_ and *J*_*max*_ were determined from these *A*/*C*_*i*_ curves according to the mechanistic model of photosynthesis (Farquhar et al., 1980). *A*/*C*_*i*_ curves were analyzed using a curve fitting utility developed by Sharkey et al. (2007) with mesophyll conductance (*g*_*m*_) constrained according to values for tobacco at 25 °C reported previously with temperature dependency incorporated from the linear relationship of *g*_*m*_ with temperature where y = −0.44 + 0.058x (Evans and Von Caemmerer, 2013).

### 2.3 Leaf nitrogen concentration

In 2016, immediately following each *A*/*C*_*i*_ curve, three 2.01cm^2^ leaf disks were destructively harvested from each leaf using a cork borer and dried until constant mass and a subset of ground tissue of known mass (3 ± 0.5 mg) was combusted with oxygen in an elemental analyzer (*Costech 4010*; *Costech Analytical Technologies*) and calibrated to %N against an acetanilide standard curve.

### 2.4 Partial least squares regression (PLSR)

Two separate PLSR model build sets were performed for *V*_*c*,max_, *J*_*max*_ and [N]. One model (Model set 1) was built using data collected during the 2016 growing season, and the second (Model set 2) was built using 75% of the data collected during the 2016 and 2017 growing seasons. Model set 1 was validated against data collected in the following year (2017). Model set 2 data was validated against the 25% of data not used for the model build (Table S1). Model training data sets consisted of pairs of modelled or measured parameters with reflectance spectra measured on the same leaf. For model validation, coefficients output from the PLSR model build were applied at each spectral waveband to collected reflectance spectra to predict the trait of interest.

All models were built following the same PLS build principles (Ollinger and Smith, 2005; Asner and Martin, 2008; Townsend et al., 2003) using previously published methods (Serbin et al., 2014) but modified for *N. tabacum*. An open-source Partial Least Squares (PLS) package (Mevik and Wehrens, 2007) in R (*The R Foundation for Statistical Computing*, *Wien*, *Austria*) was used to create the linear model of waveband coefficients by identifying latent variables (LVs) that account for trait variation in the reflectance spectra. It uses a leave-one-out cross validation approach that then makes a prediction for the out-of-sample observation (Siegmann and Jarmer, 2015). The predicted residual sum of squares (PRESS) statistic and lowest root mean square error of prediction from cross validation (RMSEPCV) were used to determine the optimal number of LVs and to prevent overfitting. The PRESS statistic determines the number of LVs to achieve minimum root mean square error (RMSE) between modelled and observed leaf traits (Wold et al., 2001). The RMSEPCV cross validates the model bias and variance (Gowen et al., 2011).

#### 2.4.1 Model set 1

For Model set 1, the *V*_*c*,max_ model was built with a training dataset of 113 measurement pairs. The collected reflectance spectra from six genotypes (Fig. 2a) were used as a training dataset for a PLSR model build with six latent variables as determined by the PRESS statistic and RMSE (Fig. S2a, c and e). The *J*_max_ PLSR model was built independently given that the double Rubisco knockdown plants (SSuD) were found to not be electron transport limited even at high CO_2_ concentrations. As such, maximal electron transport rate could not be determined for the double Rubisco knockdown measurements (SSuD) and they were removed from the *J*_max_ model build, leaving a data training set of 94 measurement pairs, with 9 latent variables (Fig. S2b, d and f). The spectral range for the *V*_*c*,max_ and *J*_*max*_ models was 500–2400 nm, with a spectral resolution of 3 nm in the visible and NIR (350–1000 nm) and 8 nm in shortwave-infrared (SWIR; 1000–2500 nm). While spectra were collected across the full range (400–2500 nm) we excluded regions below 500 nm and above 2400 nm due to noise. The %N model was built with a training data set of 131 N values measured from leaf tissue samples in the same leaves measured for *V*_*c*,max_ and *J*_*max*_. The spectral range for the %N model was also 500–2400 nm. PLSR build statistics for all 2016 models are shown in Figs. S2–4.

The models for *V*_*c*,max_, *J*_*max*_ and %N were built with varying sample sizes, according to the reliability of leaf level field gas exchange measurements and sampling. For the %N 131 data pairs were used, for *V*_*c*,max_ 114, and for *J*_*max*_ only 97, given that a model build requires a collected spectrum and accurate ‘ground truth’ measures of leaf N, *V*_*c*,*max*_ or *J*_*max*_. In 2016, a total of 132 plants were measured (132 spectra collected). One of the leaf disks for nitrogen extraction was compromised during transport to the lab and therefore discarded, leaving 131 measurement pairs to build the %N model. CO_2_ response curves made with gas exchange systems respond to plant environment and physiology over the ~30mins required per response curve, and thus have a greater margin for error. If the collected curves could not be fit for a reliable value for *V*_*c*,max_ and *J*_*max*_, they were removed from the analysis prior to any model build. For example, if stomatal conductance was shown to be limiting, or the sum of squares from being fit according to the Farquhar et al. (1980) model of photosynthesis was greater than 100, the derivation of *V*_*c*,max_ and *J*_*max*_ was deemed unreliable and removed from the analysis. Reliable modelled values of *J*_*max*_ were further reduced compared with *V*_*c*,*max*_ given the inability to define an electron transport limited state for the plants with reduced Rubisco. No data pairs with reliable ground truth measurements for %N, *V*_*c*,max_ and *J*_*max*_ were removed from analysis beyond this point for any model builds. No outliers were removed from any models.

#### 2.4.2 Model set 2

The same PLSR model builds protocol as for Model Set 1 was followed for Model Set 2. The *V*_*c*,max_ model was built with a training dataset of 186 measurement pairs of collected reflectance spectra and modelled gas exchange *V*_*c*,*max*_ values from 9 genotypes, with 15 latent variables as determined by the PRESS statistic and RMSEPCV (Fig. S5a, c and e). The *J*_*max*_ model was built independently, again with the double Rubisco knockdown measurements (SSuD) removed, leaving a data training set of 165 measurement pairs, with 15 latent variables (Fig. S5b, d, f). PLSR scores for both models are presented in Fig. S6 and model residuals in Fig. S7. The spectral range for the *V*_*c*,max_ and *J*_*max*_ models was 500–2400 nm. Newly generated PLSR coefficients were applied to the spectra from the 25% holdout validation dataset for both *V*_*c*,*max*_ and *J*_*max*_.

## 3 Results

### 3.1 Model build set 1

**Fig. 1:**
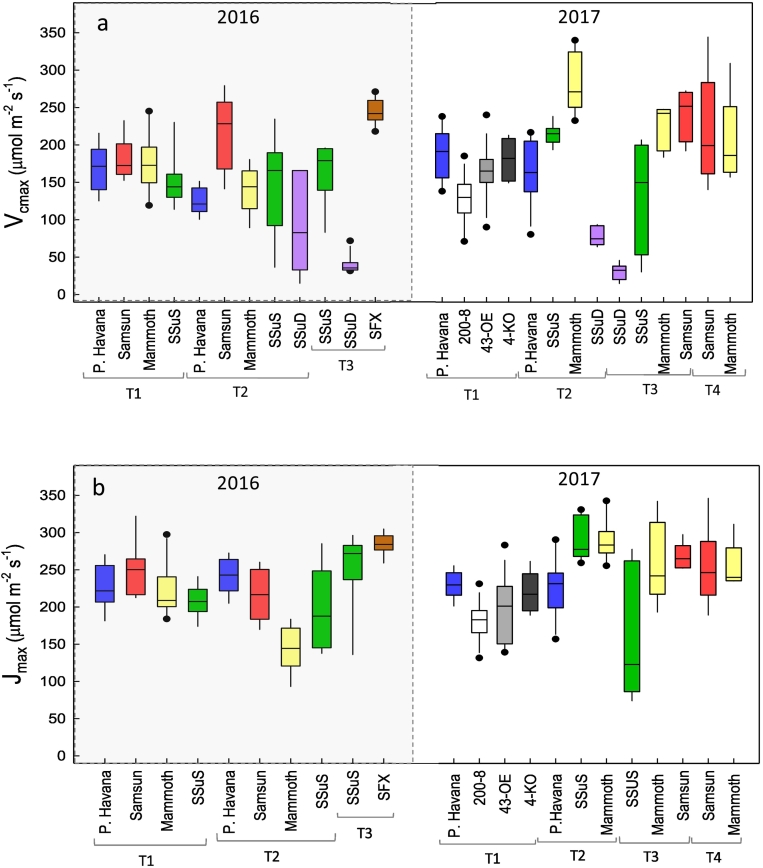
Box plots for *V*_*c*,*max*_ (a) and *J*_*max*_ (b) calculated from photosynthetic-CO_2_ response curves for tobacco plants over two growing seasons. The boxes show the interquartile range with the median as solid horizontal line. Whiskers show data outside the interquartile range but within 1.5× the interquartile range. Dots show outliers. Colors are included to assist in comparisons with Fig. 3, Fig. 5, Fig. 6, Fig. 8. (For interpretation of the references to color in this figure legend, the reader is referred to the web version of this article.)

**Fig. 2:**
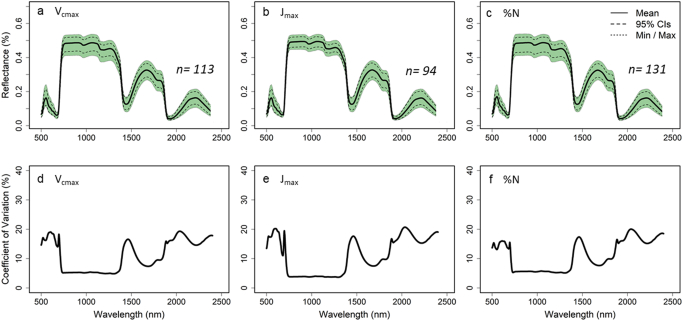
Mean, 95% confidence intervals, and minimum and maximum leaf reflectance for all leaves of *Nicotiana tabacum* used for the 2016 V_cmax_ (a), J_max_ (b) and %N (c) model builds and the co-efficient of variation across the full spectra for each model build respectively (d, e and f).

Measurements for the Model Set 1 made over the 2016 growing season (Fig. 1) represented a wide range of meteorological conditions (Fig. S1), which coupled with the different cultivars and genetic modifications, yielded a wide range of values for *V*_*c*,max_ (14.7–279.8 μmol CO_2_ m^−2^ s^−1^) and *J*_*max*_ (92.8–323.2 μmol CO_2_ m^−2^ s^−1^). To build a predictive model for *V*_*c*,*max*_, *J*_*max*_ and [N], measurements of each parameter were paired with collected reflectance spectra from the same leaves (Fig. 2).

**Fig. 3:**
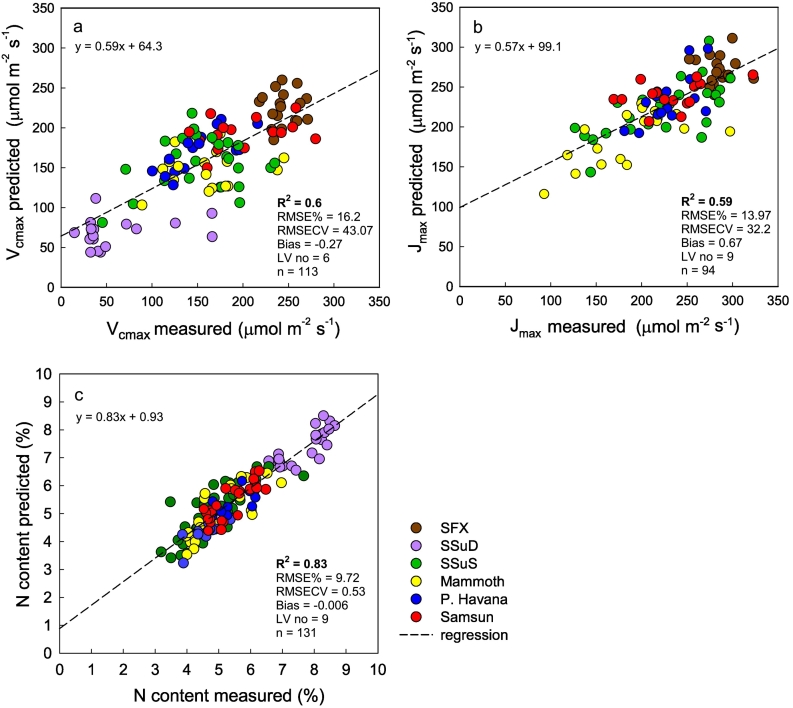
PLSR predicted from leaf spectral measurement (500–2400 nm) vs. measured using traditional techniques of *V*_*c*,max_ (a), *J*_*max*_ (b) and [N] (c) from 6 tobacco genotypes in 2016. The dashed line represents a linear regression fit to the data with statistical results are inset.

For Model Set 1, the PLSR model for *V*_*c*,*max*_ (R^2^ = 0.6; Fig. 3a), *J*_*max*_ (R^2^ = 0.59; Fig. 3b), and [N] (R^2^ = 0.83; Fig. 3c) all showed strong positive correlations with the measured values. Leaf [N] was very high in the SSuD double Rubisco knockdown leaves which had the lowest *V*_*c*,max_ (Fig. 3). Fig. S2 shows the number of latent variables (LV's) chosen for each model build, defined as the LV number that minimizes the predicted residual error sum of squares (PRESS) statistic and root mean square error of prediction from cross validation (RMSECV) from the predictions vs. sample observation fit. In model set 1, six LV's were used for the *V*_*c*,max_ build, while nine LV's were used for *J*_max_ and [N]. PLSR scores for LV's and model residuals are shown in Figs. S3 and S4, respectively, with residuals evenly centered around zero for all model builds, confirming the suitability of a linear regression model.

**Fig. 4:**
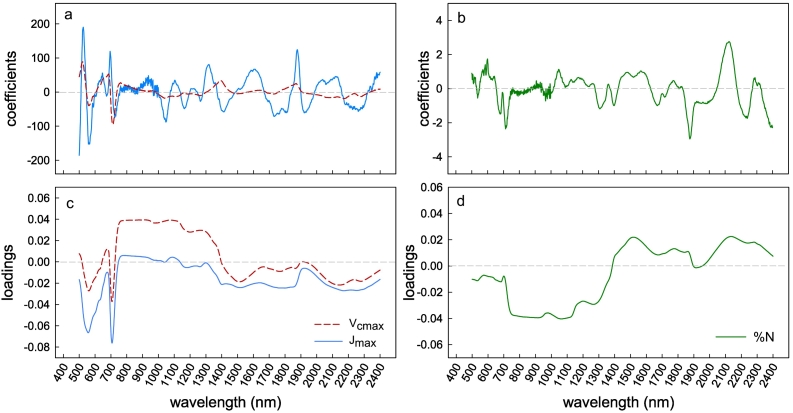
Model build set 1 spectral-specific coefficients for *V*_*c*,max_ and *J*_*max*_ (a) and %N [N] (b), with model loadings for *V*_*c*,*max*_ and *J*_*max*_ (c) and %N (d).

Model coefficients generated from the model fit center around zero in all model builds (Figs. 4a and b). Model loadings indicate the impact of spectral regions on the model build.

Where loading values are closer to zero, they have a lower impact on the model build and the more the values deviate from zero indicate greater influence on the PLSR predictions. *V*_*c*,*max*_ model loadings across the spectra (Fig. 4c) showed stronger correlation in the chlorophyll bands (450–550 nm and 640–680 nm), across the red edge (680–730 nm), and across the NIR (900–1400 nm). In the short wave infra-red region (177–2400 nm) loading values had a lesser impact than in the visible and near infra-red ranges. The same chlorophyll bands were highly loaded in the *J*_max_ model, yet the NIR region (800–1400 nm) and SWIR (1700–2400) had a lower loading weight (Fig. 4c). Percentage [N] model loadings were strong across the NIR spectra (700–1300 nm) and from 1500 to 2400 nm in the SWIR, with areas of lower loading weight between 1300–1400 and 1900–2000 nm (Fig. 4d).

**Fig. 5:**
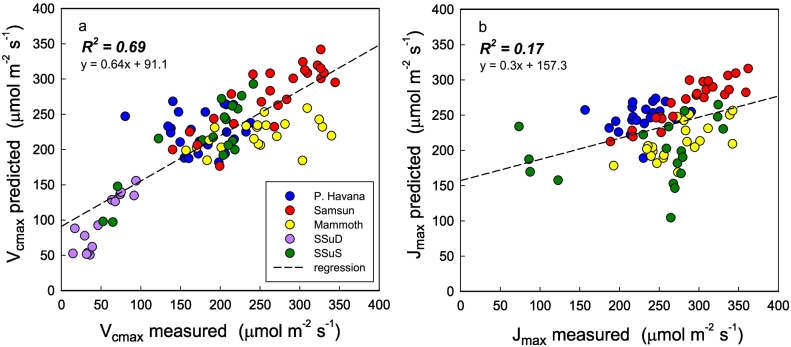
Validation of model build set 1 for *V*_*c*,max_ and *J*_max_ using the same genotypes measured in 2017. The regression equation and R^2^ are inset for each graph.

**Fig. 6:**
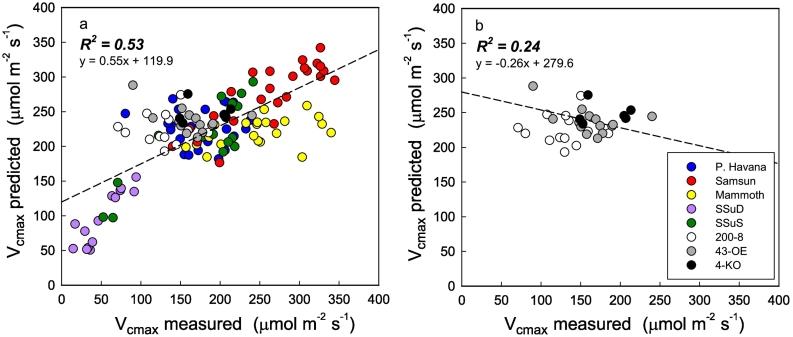
PLSR coefficients from 2016 model build shown in Fig. 3 applied to reflectance spectra collected in 2017 to predict *V*_*c,*max_ in 8 *Nicotiana tabacum* genotypes (a). The 3 newly added transgenic genotypes in 2017 are separated from the dataset, and the same PLSR coefficients applied to predict *V*_*c*,max_ in those genotypes alone (b).

Spectral measurements were coupled with gas exchange measurements from leaves at varying growth stages and meteorological conditions during 2017 in the same cultivars to test the model build from 2016 (Fig. 5). With PLSR coefficients applied to these collected leaf reflectance spectra, Model set 1 correlated strongly for measured *V*_*c*,max_ (R^2^ = 0.69) but not for *J*_*max*_ (R^2^ = 0.17) in 2017 (Fig. 4). However, photorespiratory bypass and modified photoprotection transgenic lines (200-8, 43-OE and 4-KO) that were not grown in 2016 reduced the predictive power of *V*_*c*,max_ Model set 1 when added to the validation dataset (Fig. 6a). Predicting *V*_*c*,max_ for all 2017 genotypes using Model set 1 resulted in lower R^2^ values of 0.53 (Fig. 6a). Applying the PLSR coefficients solely to the three newly added 2017 transgenic cultivars showed no predictive power for *V*_*c*,max_ (Fig. 6b).

### 3.2 Model build set 2

**Fig. 7:**
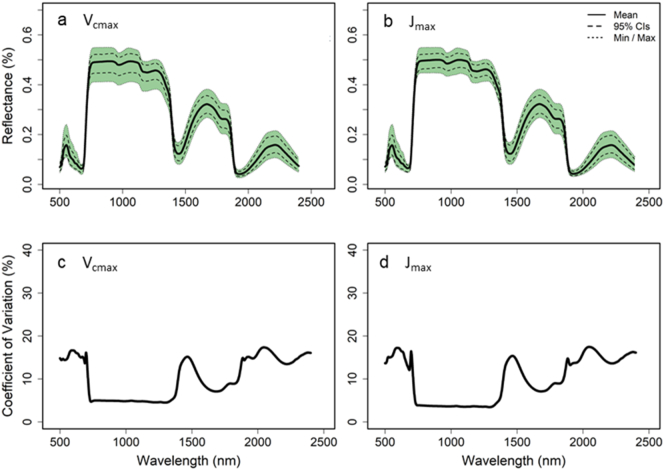
Mean, 95% confidence Intervals, and minimum and maximum leaf reflectance Model set 2 for V_cmax_ (a) and J_max_ (b) and the co-efficient of variation for the full spectra for both models respectively (c and d).

**Fig. 8:**
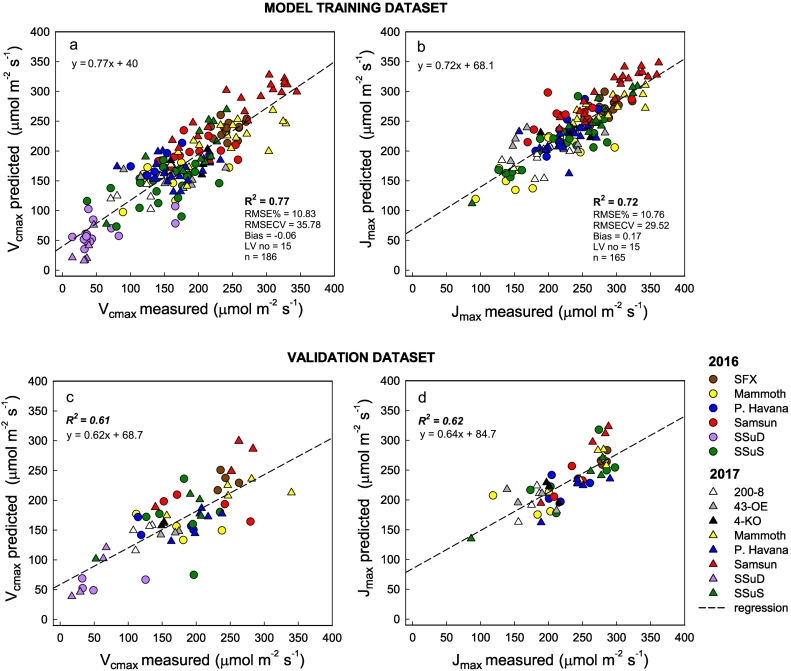
Measured versus predicted PLSR values of *V*_*c*,*max*_ (a) and *J*_*max*_ (b) from PLSR models built with 75% of data collected in 2016 and 2017, randomly selected for model training (Model set 2). Model build statistics are presented in Figs. S5–7.

Model build set 2 used the data collected in 2016 and 2017 with 75% of the spectra from both years (Fig. 7) used to train a second PLSR model to test against the remaining 25%. The new model showed a strong relationship for *V*_*c*,max_ (R^2^ = 0.77, y = 0.77x + 40; Fig. 8a) and *J*_*max*_ (R^2^ = 0.72, y = 0.72x + 68.1; Fig. 8b). Both models were built with 15 latent variables (LV's) through determination of the PRESS statistic (Fig. S5). PLSR scores are shown in Fig. S6 and model residuals had an even spread around zero (Fig. S7).

**Fig. 9:**
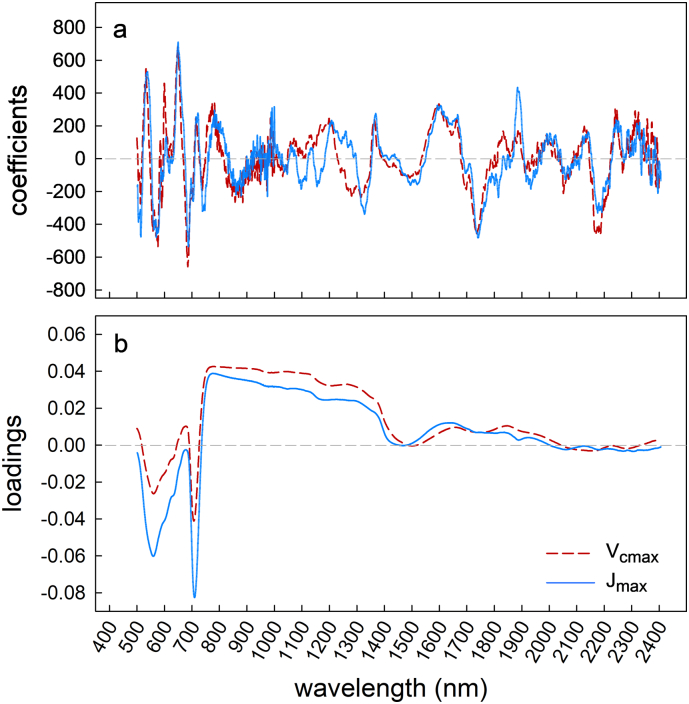
Model build set 2 generated coefficients (a) and loading weights (b) for *V*_*cmax*_ and *J*_*max*_.

The RMSEP CV was 35.78 and 29.52 μmol m^−2^ s^−1^ and model bias was −0.06 and 0.17% for *V*_*c*,max_ and *J*_*max*_, respectively. With 25% of the data randomly selected and held back for cross validation by application of the PLSR coefficients from the new model (Fig. 9a), both *V*_*c*,max_ and *J*_*max*_ were faithfully predicted from the reflectance spectra where R^2^ for *V*_*c*,max_ was 0.61 (Fig. 8c) and for *J*_*max*_ was 0.62 (Fig. 8d). Model loadings for model set 2 (Fig. 9b) follow similar patterns to model set 1 (Fig. 4c), with the exception that *J*_max_ has higher loadings in the NIR region (800–1400 nm).

## 4 Discussion

This research addressed whether the PLSR method can be applied to genetically modified crops to rapidly and accurately predict *V*_*c*,max_ and *J*_*max*_ (Prediction 1) and to assess interannual variability in photosynthetic performance (Prediction 2). We further sought to test whether spectra-based *V*_*c*,max_ and *J*_*max*_ models would be independent of spectra-leaf nitrogen relationships rather than arising from the close inter-relationships between *V*_*c*,max_ and *J*_*max*_ with leaf nitrogen (Prediction 3).

The PLSR models were able to predict significant variation in *V*_*c*,max_ and *J*_*max*_ imposed by genetic modification and measured across growing seasons, supporting Prediction 1. A PLSR model built using 2016 data (Model set 1) resulted in a strong ability to predict *V*_*c*,*max*_ in 2017 but not *J*_*max*_, supporting Prediction 2 for *V*_*c*,*max*_, but not for *J*_*max*_. Further, the PLSR Model set 1 was only successful in predictions of *V*_*c*,*max*_ and *J*_*max*_ for genotypes used to build the model in 2016, i.e., the model was unable to predict either *V*_*c*,*max*_ or *J*_*max*_ for genotypes unique to 2017 using a model built with data from 2016 (Fig. 6a). Finally, the model worked for all species, including a genetically modified genotype showing the lowest *V*_*c*,max_ yet the highest [N], reverse for all other genotypes. That the model faithfully predicted both *V*_*c*,*max*_ and *J*_*max*_ for this genotype suggests that the PLSR model is not merely using leaf [N] as a proxy of for photosynthetic physiology, which supported Prediction 3.

Measurements represented a wide range of photosynthetic capacity and leaf nitrogen concentrations, however the full strength of the model was only realized when built using data that captured the full range of interannual and genetic variation (Fig. 8). The application of our 2016 model to the 2017 dataset for the same species but with three additional transgenic lines (Fig. 6) showed that the model may only be applicable to the varieties used to build the model, regardless whether wild-type or genetically modified. PLSR coefficients from Model set 1 predicted 2017 measurements well (Fig. 4) but only for *V*_*c*,max_ and for the genotypes present in both years.

The inability of model build set 1 to predict *J*_*max*_ in 2017 may be linked with the underlying processes represented by *J*_*max*_ compared with *V*_*c*,*max*_. The total amount of the enzyme Rubisco that is present and metabolically active determines *V*_*c*,*max*_ (Bernacchi et al., 2001; Portis, 2003; Suzuki et al., 2009). While there is substantial uncertainty surrounding the mechanism being used to predict *V*_*c*,*max*_, there is no interannual variation in the structure and function of Rubisco. Therefore, the performance of the 2016 model in predicting *V*_*c*,*max*_ in 2017 is not unexpected. However, *J*_*max*_ represents the coordination of a complex series of reactions involving many proteins integrated into the thylakoid membrane in the chloroplast (Farquhar et al., 1980; von Caemmerer, 2000). Furthermore, the estimation of *J*_max_ may not necessarily reflect rates of electron transport as more recent research suggests *J*_*max*_ may be constrained by metabolic reactions involved in the regeneration of RuBP in the photosynthetic ‘dark’ reactions (Raines, 2003; Lefebvre et al., 2005). Therefore, the more complex metabolic processes associated with *J*_max_ likely leads to its poor performance compared with *V*_*c*,*max*_ (Fig. 5b). A similar challenge is observed when trying to model photosynthesis using *J*_*max*_ for plants grown under different environmental conditions (Bernacchi et al., 2003; Köhler et al., 2016). The model built with 2016 and 2017 data (Model set 2) predicted *V*_*c*,*max*_ and *J*_*max*_ better (Fig. 8a) than a model built with only 2016 data (Fig. 3). R^2^ values were higher and RMSE was reduced in model build set 2 for both *V*_*c*,max_ (from 16.2 to 10.8%) and *J*_*max*_ (14.0 to 10.1%). Model bias also decreased for both *V*_*c*,*max*_ (from −0.27 to −0.06%) and *J*_*max*_ (from 0.67 to 0.17%).

These results suggest that the importance of including environmental variation when building PLSR models (Wu et al., 2017) is matched by the importance of incorporating genetic variation. Combined over both years, a total of nine genotypes each expressing different photosynthetic phenotypes were used in this analysis and the model only performed well when validated against the same genotypes used to build the model. Whether a PLSR model built using dozens or hundreds of genotypes can reliably predict photosynthetic physiology for a unique genotype still needs to be resolved.

The dependence of *V*_*c*,max_ and *J*_*max*_ on temperature is well documented (Farquhar et al., 1980; von Caemmerer, 2000; Bernacchi et al., 2001, Bernacchi et al., 2003). Therefore, the gas exchange measurements were collected at the same temperature as leaf spectral measurements to remove temperature variation between the measured and modelled parameters. Normalizing to a standard temperature might lead to improved regressions but would challenge the application of this technique under field-ambient conditions where temperatures constantly fluctuate.

PLSR model loadings identify regions of the spectrum significant for the trait of interest, by identifying maximum variations common to all spectra in the dataset (Wold et al., 2001; Serbin et al., 2012). Highly loaded regions are linked with physiological indicators, which suggest that using PLSR to mathematically analyze reflectance spectra may be more than purely empirical. The PLSR model loadings across the spectra for *V*_*c*,*max*_ and *J*_*max*_ for build 1 show strong correlations between 450 and 680 nm and across the ‘red-edge’ (Fig. 4c). Leaf reflectance spectra between 450 and 680 nm is influenced by photosynthetic pigments (Gates et al., 1965; Knipling, 1970; Rouse Jr et al., 1974) and carotenoid content (Gamon et al., 1992). Similarly, the ‘red-edge’ spectral region (680–730 nm), in which leaf reflectance greatly increases as light in the near infra-red region is no longer absorbed by chlorophyll (Woolley, 1971; Horler et al., 1983) has been shown to correlate with photosystem II function (e.g., Fv/Fm; Zarco-Tejada et al., 2000). These same spectral regions have shown similar loading for photosynthetic PLSR models of other crop species (Serbin et al., 2012; Yendrek et al., 2016; Silva-Perez et al., 2017). The lower loading weights in the *J*_*max*_ model compared with *V*_*c*,*max*_ model in the NIR region (Fig. 4c) are consistent with previous results (Serbin et al., 2012). Model build 2 (Fig. 8) showed a very similar pattern for loading weights (Fig. 9).

While model loadings are generally consistent with previous reports and suggest links with physiological controls, the underlying mechanism is unclear that PLSR models utilize to predict *V*_*c*,*max*_ and *J*_*max*_. While leaf [N] cannot be directly measured by spectral analysis, we show that it can be accurately predicted by PLSR analysis (Fig. 3c) consistent with other studies (Kattge et al., 2009; Serbin et al., 2012; Rogers, 2014; Walker et al., 2014; Yendrek et al., 2016; Dechant et al., 2017; Silva-Perez et al., 2017). Given that photosynthetic enzymes, predominantly Rubisco, account for a large proportion of leaf nitrogen, under favorable environmental conditions photosynthetic rate per unit leaf area (photosynthetic capacity) increases linearly with leaf nitrogen content (Field and Mooney, 1986; Evans, 1989; Poorter et al., 1990; Nakano et al., 1997; Reich et al., 1998). Yet, in the SSuD Rubisco antisense line this relationship is reversed – the SSuD lines have, by far, the lowest *V*_*c*,*max*_ and the highest [N], similar to other studies using tobacco with reduced Rubisco (Quick et al., 1991; Masle et al., 1993). Despite this genotype differing from all others, the *V*_*c*,*max*_ and [N] models faithfully predict each phenotype independently (Fig. 3a and c). This suggests that, despite previous research suggesting the PLSR model is dominated by a nitrogen signal (Kattge et al., 2009; Rogers, 2014; Walker et al., 2014; Dechant et al., 2017), the model estimates of the traits are independent. While the results are unable to elucidate a key mechanistic understanding of what the PLSR method is measuring, they are both a valuable step in understanding that the relationship is not simply a proxy for leaf nitrogen and that this method can be very useful in phenotyping both wild-type and genetically modified plants despite the lack of mechanistic understanding.

The model shows strong predictive ability over a wide range of environmental conditions for plants showing a range of phenotypes. Additional experiments using genotypes with distinct alterations to the photosynthetic pathway and/or related metabolic pathways can be further exploited to refine the mechanism associated with this technique. For example, Rubisco active site inhibitors, which reduce the catalytic activity of Rubisco, thereby reducing *V*_*c*,*max*_ without changing the concentration of Rubisco, may provide more insight into the mechanisms behind the PLSR results. Machine learning analysis using plants with genetic modifications that extend beyond photosynthesis may elucidate spectral regions driving PLSR predictions to provide further physiological interpretation.

The results presented here also suggest that caution must be exercised with reflectance-based analysis to infer crop productivity or health for genetically modified plants. Introduction of transgenic modifications to the photosynthetic pathway for the SSuD tobacco line led to shifts in known relationships between leaf chemistry and photosynthetic capacity. Using many established reflectance-based approaches, particularly related to simple indices (e.g., PRI, NDVI), would suggest the high leaf [N] for SSuD might predict higher biomass. Therefore, the widely held and supported view that photosynthesis and leaf N content are highly correlated (Evans, 1989) for wild-type species would suggest photosynthetic capacity and productivity of established techniques may not apply to genetically modified plants.

The full extent of current and potential opportunities to improve photosynthesis in plants extends well beyond the strategies employed in this analysis (Ort et al., 2015). Continued use and development of high throughput techniques using novel genotypes holds potential for further insights into the mechanisms behind spectral shifts and how they relate to photosynthetic physiology. Furthermore, it may prove to be a useful tool as novel breeding strategies are realized. Extending this model to incorporate spectra collected from imaging systems, thereby removing the need for leaf-clip based measurements, may increase throughput capabilities by orders of magnitude and provide critical information related to canopy-scale variance in photosynthetic physiology. The need to understand agronomic ecosystem functioning has led to substantial efforts to measure productivity over large spatial scales. Using solar induced fluorescence (e.g., Miao et al., 2018) to quantify GPP can lead to a better understanding of ecosystem functioning. However, efforts to improve understanding of ecosystem function extend beyond in situ measurements of photosynthesis and towards understanding the underlying physiology necessitating techniques such as those described here.

## 5 Conclusions

We show the spectral PLSR method can be applied to genetically modified crops to rapidly and accurately predict photosynthetic capacity. However, models lose predictive power when used interannually on new genetic material not included in model builds. Despite the strengths of the model, the results suggest a need for repopulation of PLSR models annually when dealing with discreet variation in photosynthetic capacity between genotypes of a single species in crop trials. However, the extent that models need to be repopulated in time and space for hyperspectral PLSR models is still uncertain. The need for repopulating the model may also apply when using hyperspectral PLSR to compare photosynthetic physiology between geographical regions or when environmental conditions change dramatically over time. We also deconvolute the relationship between photosynthetic capacity and leaf nitrogen content, which speaks to the potential of full spectral analysis for elucidating biochemical mechanism from spectral reflectance with further work, at any scale.

Our results suggest that a link between remote sensing and photosynthetic physiology can be applied to agricultural species. This provides opportunity to better parameterize agricultural models and to identify variation in photosynthetic physiology for breeding efforts. As future food production will almost certainly depend on genetically modified crops (Ort et al., 2015), we also show that novel techniques will continue to be useful as the agricultural landscape continues to change.

## Appendix A Supplementary data

Supplementary material Image 1

Model set 1 – Coefficients and loading. Model coefficients generated by the PLSR statistics to predict the trait of interest (V_cmax_, J and N) for model build 1 (Fig. 4a & b). Model loadings indicate the relative importance of the given spectral waveband in the model builds for each trait (Fig. 4c & d). Model set 1 – Nitrogen and spectra. Here the reflectance spectra (average of 6 measurements on a single leaf) collected in 2106 with Fieldspec 4 (*Analytical Spectral Devices* - *ASD*, *Boulder*, *CO USA*) are presented alongside leaf N% collected from destructive sampling. These data were used to train the PLSR model for leaf N in Fig. 3c. Model set 1 – Raw spectra, V_cmax_ and J_max_. Collected reflectance spectra (average of 6 measurements on a single leaf) in 2016 with Fieldspec 4 (*Analytical Spectral Devices* - *ASD*, *Boulder*, *CO USA*) alongside modelled V_cmax_ and J. These data were used to train and build the PLSR models for V_cmax_ and J in Fig. 3a & b. Model set 2 - Coefficients and loading. Model coefficients generated by the PLSR statistics required to predict V_cmax_ and J for model training data in model build 2, shown in Fig. 9b. Model loadings indicate the relative importance of the given spectral waveband in this second model build (Fig. 9a). Model set 2 – Training data (75%) Raw spectra, V_cmax_ and J_max_. Collected reflectance spectra in 2016 and 2017 (average of 6 measurements on a single leaf) with Fieldspec 4 (*Analytical Spectral Devices* - *ASD*, *Boulder*, *CO USA*) alongside modelled V_cmax_ and J. These data were used to train and build the PLSR models for V_cmax_ and J in Fig. 8a & b. Model set 2 – Validation spectra (25%). Collected reflectance spectra in 2016 and 2017 (average of 6 measurements on a single leaf) with Fieldspec 4 (*Analytical Spectral Devices* - *ASD*, *Boulder*, *CO USA*) used to validate model build 2 in Fig. 8c & d. Image 2
