# Supplementary material for: High-throughput field phenotyping using hyperspectral reflectance and partial least squares regression (PLSR) reveals genetic modifications to photosynthetic capacity
Source: Remote Sens Environ. 2019 Sep 15;231:111176. doi: 10.1016/j.rse.2019.04.029 (PMC6737918; doi:10.1016/j.rse.2019.04.029)
Supplement: Supplementary file 1 — Supplementary material [file mmc1.pdf]

Supplementary Figures

Table S1 Samples numbers used in model builds for 2016 model (model build set 1), and the combined 2016-2017 model (model build set 2). Sample numbers vary for each model build depending on successful analysis of ground truthing gas exchange (V_{cmax} and J_{max}) and leaf sampling (%N) data to match collected reflectance spectra.

MODEL BUILD SET 1		Samples no. used for model build 2016		
**Year**	**Genotype**	**V_{cmax}**	**J_{max}**	**%N**
2016	SFX	18	18	0
	SSuD	19	0	23
	SSuS	28	28	36
	Mammoth	18	18	24
	Petite Havana	15	15	24
	Samsun	15	15	24
Totals 2016		***113***	***94***	***131***

		Samples no. collected in 2017	
**Year**	**Genotype**	**V_{cmax}**	**J_{max}**
2017	SSuD	14	0
	SSuS	17	17
	Mammoth	21	21
	Petite Havana	23	23
	Samsun	24	24
	200-8	13	13
	4-KO	7	7
	43-OE	14	14
Totals 2017		***133***	***119***

MODEL BUILD SET 2		
Total 2016 &2017	***246***	***213***
75% 2016 &2017 model build set	186	165
25% 2016&2017 validation set	60	48

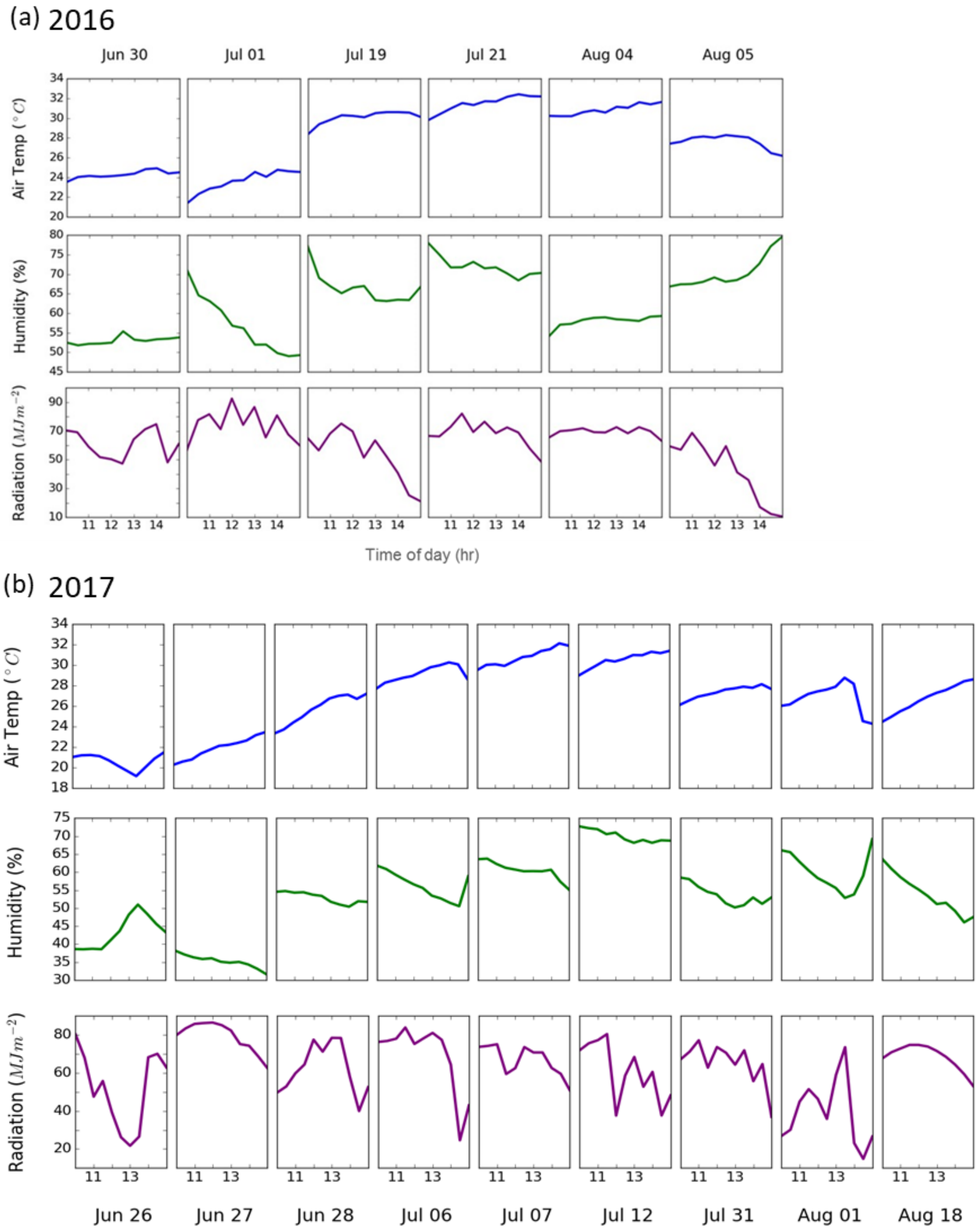

Figure S1

Air temperature, humidity and incoming solar radiation for 2016 (a) and 2017 (b) measurement days and time periods around solar noon (10am-3pm). Meteorological data was measured with a Viasala HMP45C temperature and humidity probe (Campbell Scientific, UT, USA) and CNR1 Net radiometer (*Kipp & Zonen, Netherlands*) mounted on a meteorological tower ~300m from the experimental site at the University of Illinois Energy Farm Facility in Urbana, Illinois.

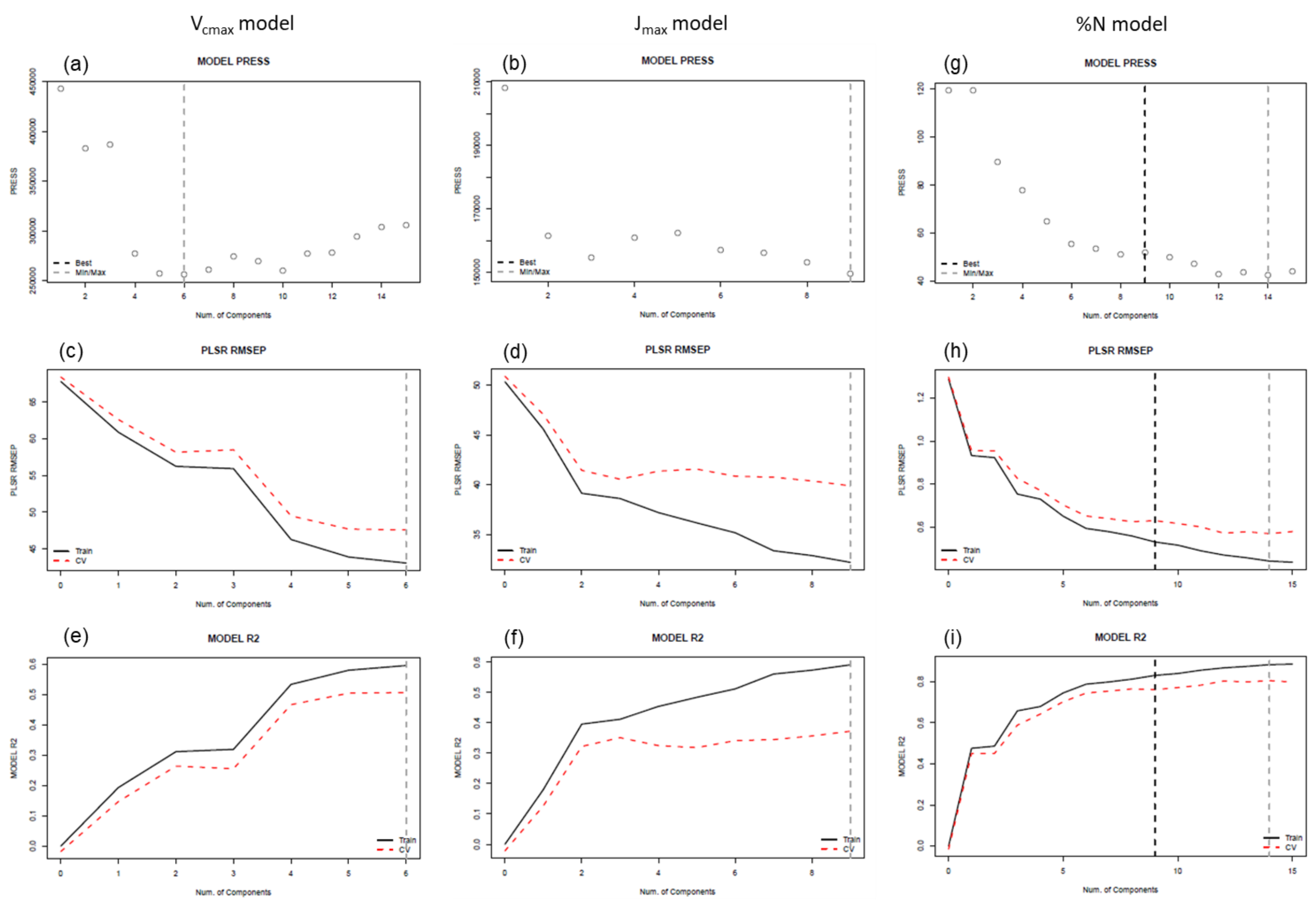

Figure S2

Model component diagnostics for the 2016 V_{cmax}, J_{max} and %N PLSR model builds. The PRESS statistic for selection of principle component number (a,b &c) , the difference between the root mean square error (d, e &f), and R^2 of the training dataset and the cross validation (g,h,&i) for each model build respectively

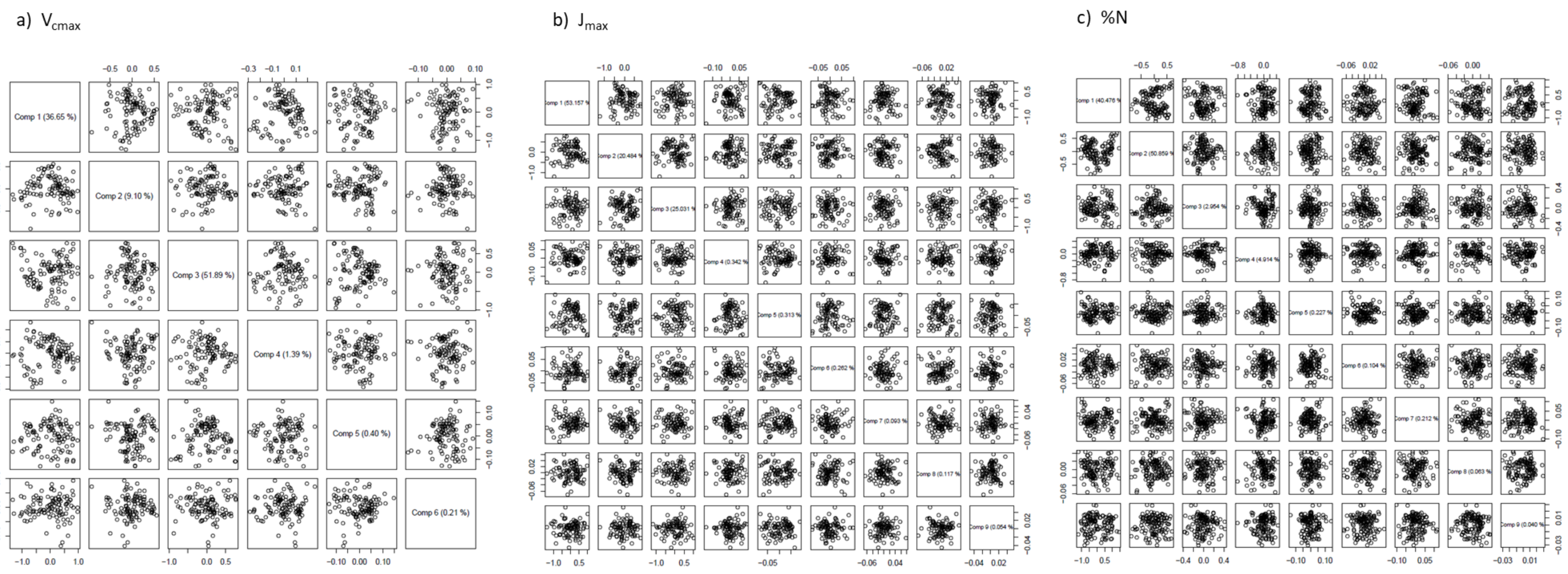

Figure S3

PLSR scores for latent variables for model build set 1: the 2016 V_{cmax} (a), J_{max} (b) and %N (c) model build.

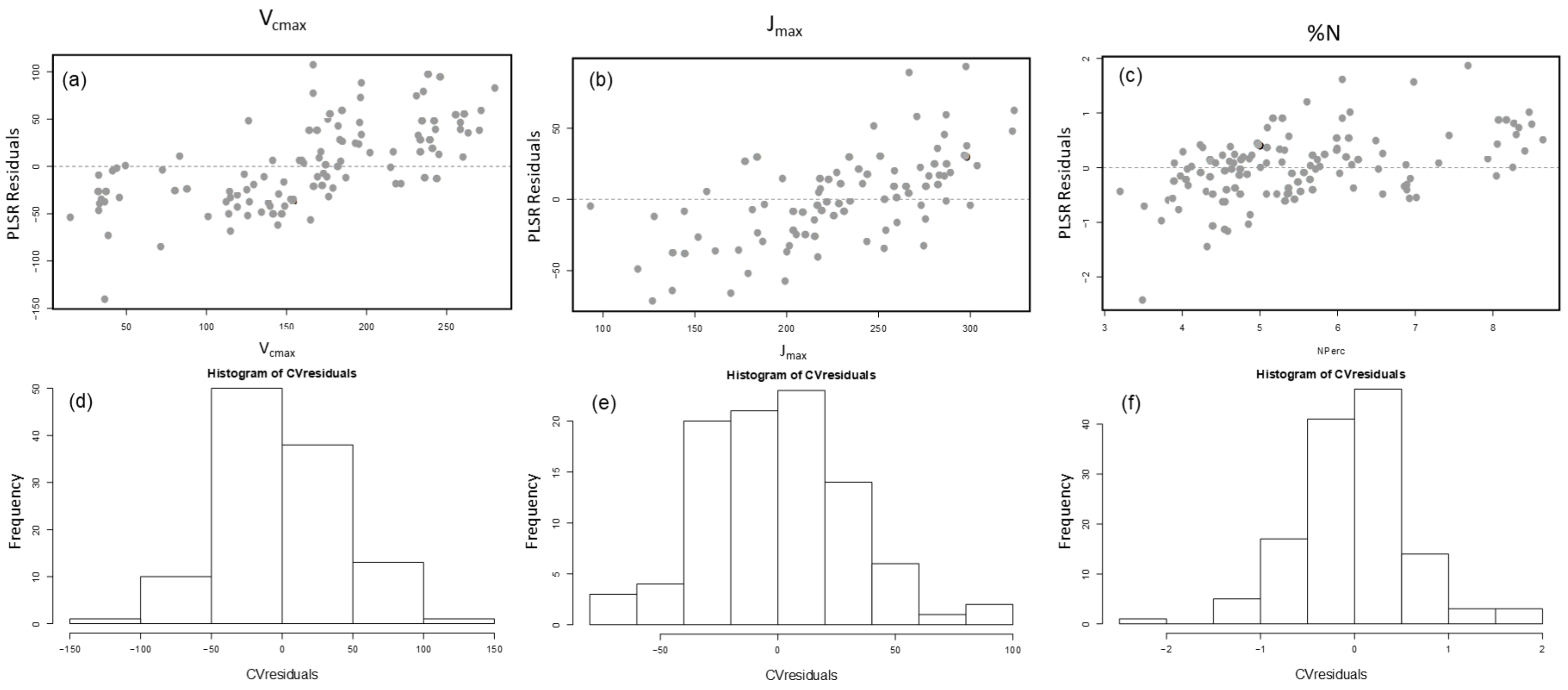

Figure S4

PLSR model residuals for Model build set 1: the 2016 V_{cmax} (a) J_{max} (b) and %N (c) models, with histograms of cross validation residual frequency for each model respectively (d, e and f).

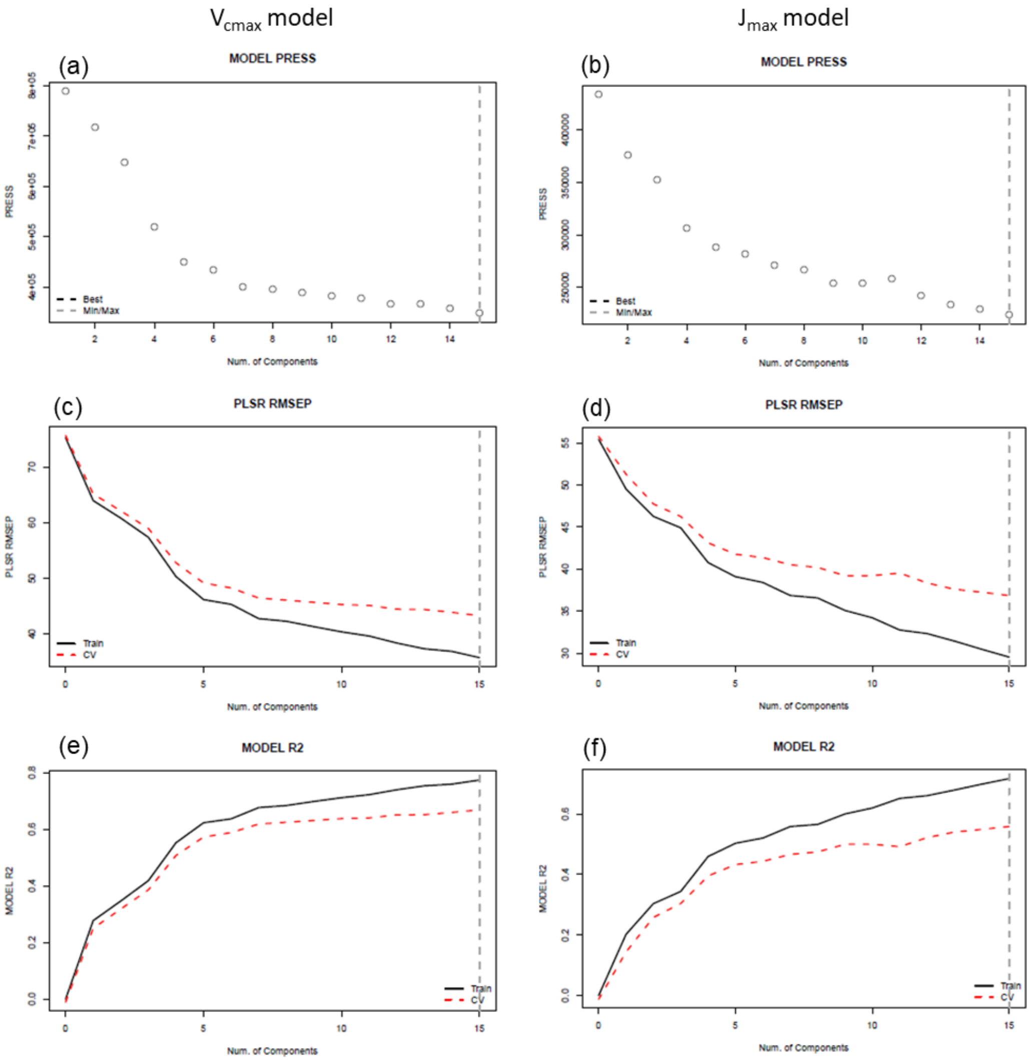

Figure S5

Model component diagnostics for model build set 2: the 2016 and 2017 combined, V_{cmax} and J_{max} PLSR model builds. The PRESS statistic for selection of principle component number (a & b), the difference between the root mean square error (c & d) and R^2 of the training dataset and the cross validation (e & f) for each model build respectively.

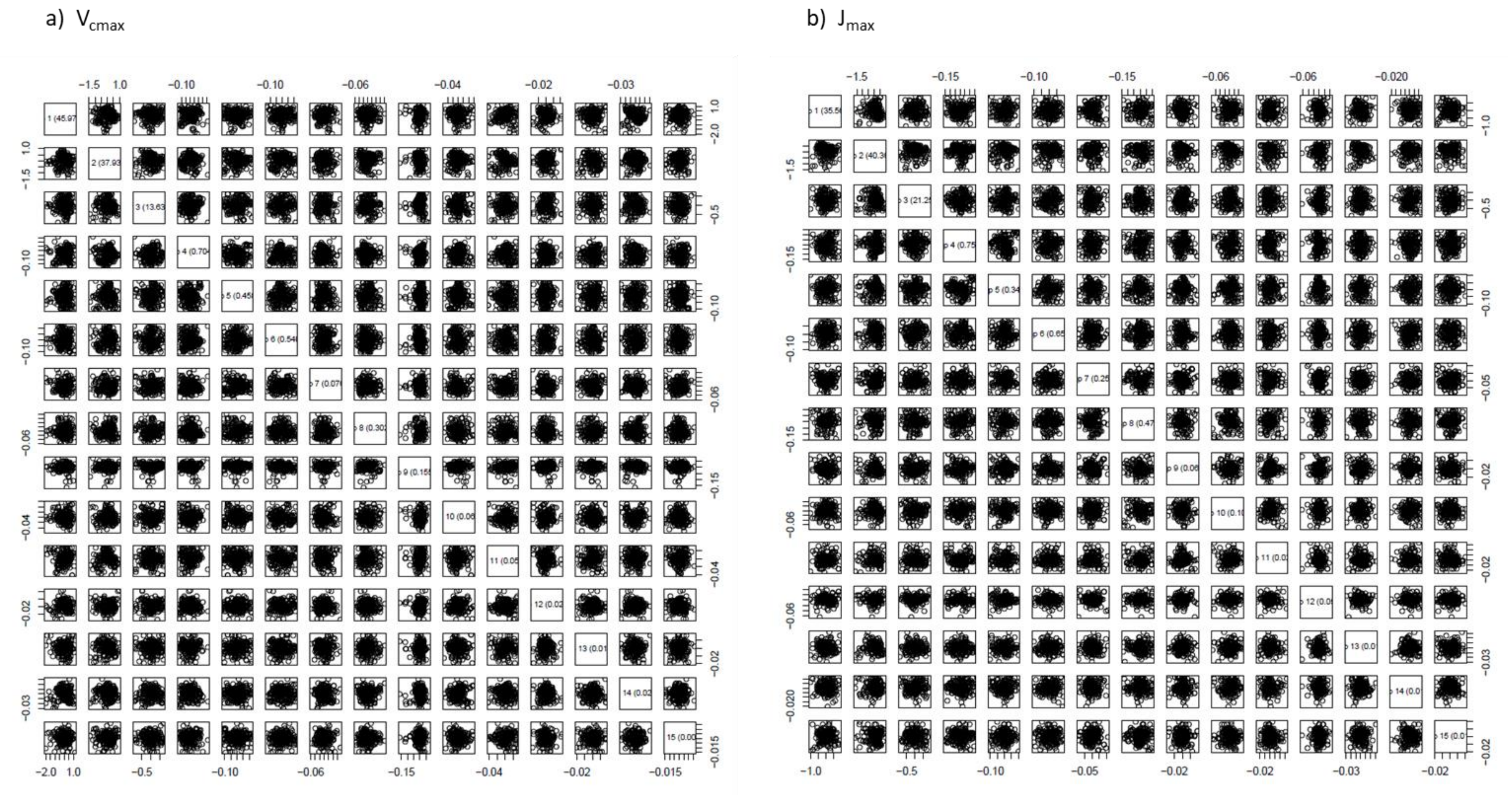

Figure S6

PLSR scores for latent variables for model build set 2: V_{cmax} (a) and J_{max} (b) 2016 and 2016 combined model build.

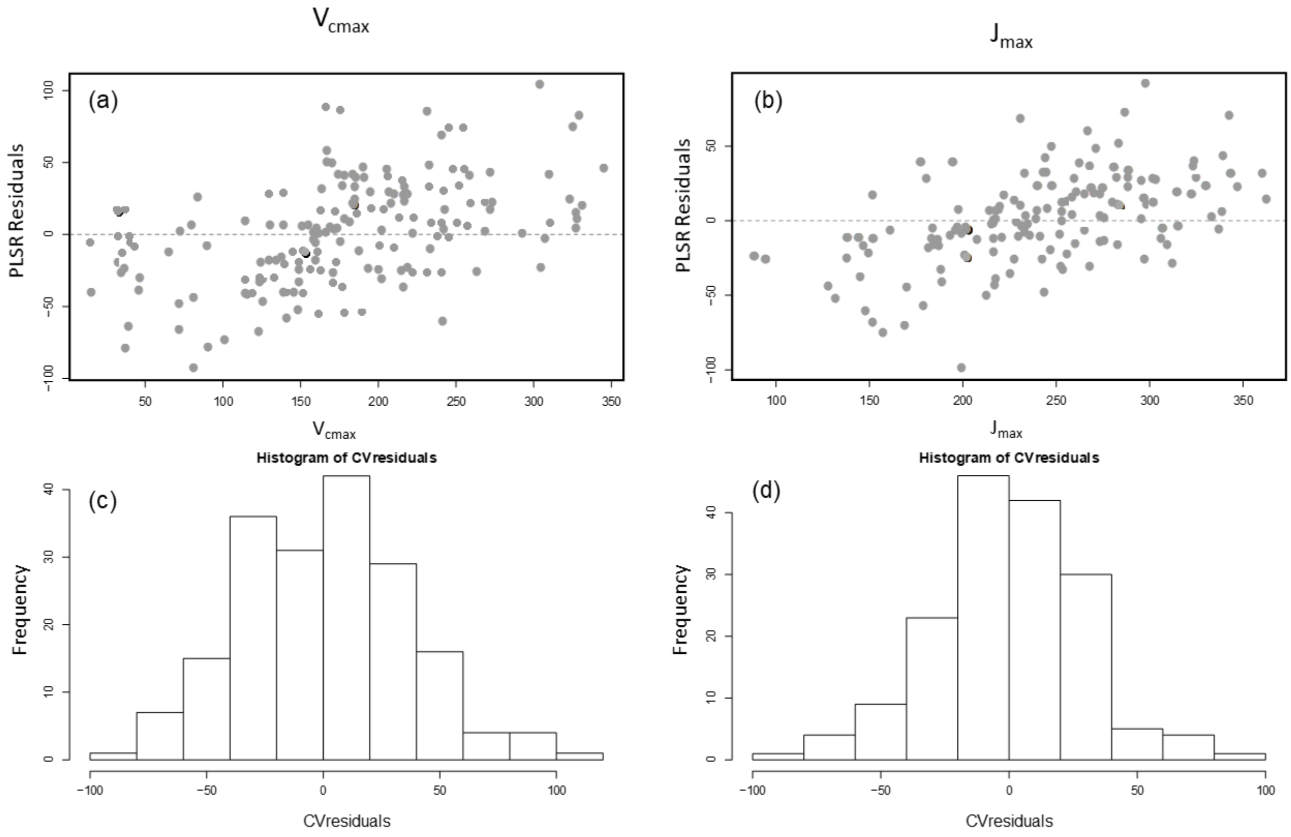

Figure S7

PLSR model residuals for Model build set 2: the 2016 and 2017 combined, V_{cmax} (a), J_{max} (b) models, with histograms of cross validation residuals for each model build respectively (c and d).
